# The pan-African natural products library compounds oleanolic acid, poinsettifolin B, and rhuschalcone III disrupt SARS-CoV-2 spike-host ACE2 interactions and SARS-CoV-2 replication

**DOI:** 10.1186/s40360-026-01264-3

**Published:** 2026-10-21

**Authors:** Mathieu J. M. Tjegbe, Pascal Amoa Onguéné, Boris D. Bekono, Jude Y. Betow, Conrad V. Simoben, Joel Cassel, Joseph M. Salvino, Luis J. Montaner, Kerstin Andrae-Marobela, Fidele Ntie-Kang, Ian Tietjen

**Affiliations:** 1 Center for Drug Discovery, Faculty of Science, University of Buea, P. O. Box 63, Buea, Cameroon; 2 Department of Chemical Biology, Faculty of Science, University of Bertoua, P. O. Box 416, Beroua, Cameroon; 3 University Institute of Wood Technology, University of Yaoundé 1, Mbalmayo Campus, Mbalmayo, Cameroon; 4 Department of Physics, Ecole Normale Supérieure, University of Yaoundé 1, P. O. Box 47, Yaoundé, Cameroon; 5 Department of Chemistry, Faculty of Science, University of Buea, P. O. Box 63, Buea, Cameroon; 6 Structural Genomics Consortium, University of Toronto, Toronto, ON M5G 1L7 Canada; 7 Department of Pharmacology & Toxicology, University of Toronto, Toronto, ON M5S 1A8 Canada; 8 The Wistar Institute, Philadelphia, PA 19104 USA; 9 Department of Biological Sciences, University of Botswana, Gaborone, Botswana; 10 Institute of Pharmacy, Martin Luther University Halle-Wittenberg, Halle (Saale), Germany; 11 School of Business, Arts, and Sciences, Delaware Valley University, Doylestown, PA 18901 USA

**Keywords:** Natural products, SARS-CoV-2, Viral entry, Antivirals, p-ANAPL

## Abstract

Natural product (NP)-based chemical libraries are a rich source of antiviral compounds that can serve as the basis for new therapeutic leads to treat viral diseases, particularly for individuals lacking sustained access to existing vaccines and therapeutics. To identify new NP-based candidate inhibitors of SARS-CoV-2 cellular entry and viral replication, we screened the pan-African Natural Products Library (p-ANAPL), a collection of physical pure compounds obtained from African medicinal plants, for NPs that can disrupt the in vitro interaction of the SARS-CoV-2 Spike receptor-binding domain (RBD) with its host ACE2 entry receptor. This screen identified three compounds – oleanolic acid, poinsettifolin B, and rhuschalcone III – which disrupt RBD/ACE2 interactions with half-maximal inhibitory concentrations (IC_50_s) of 0.5–2.4 µM but do not disrupt an unrelated PD-1/PD-L1 host ligand/receptor binding pair. Oleanolic acid and rhuschalcone III, but not poinsettifolin B, additionally inhibited SARS-CoV-2 replication in Vero cells without cytotoxicity at low micromolar concentrations. Computational modelling indicated that all three compounds interact with numerous RBD and ACE2 residues, including a subset conserved across SARS-CoV-2 variants of concern. Taken together, we identify three NPs that selectively interfere with factors involved in SARS-CoV-2 entry and/or viral replication, representing new antiviral leads for COVID-19 management in resource-limited areas.

## Introduction

While SARS-CoV-2, the causative agent of COVID-19, has become increasingly managed in much of the world, access to SARS-CoV-2 vaccines and therapeutics in many low- and middle-income countries (LMICs) remains limited or inconsistent [1–3]. Due to this inaccessibility, as well as limited access to Western medicines more generally, many people in LMICs rely on local medicinal plants for their primary healthcare [4]. Identifying natural products (NPs), or naturally produced chemical compounds, that are found in medicinal plants and inhibit SARS-CoV-2 replication is therefore important toward developing locally accessible options for management of COVID-19 and potentially other coronavirus-driven infections in the absence of sufficient availability of Western vaccines and therapeutics.

The pan-African Natural Products Library (p-ANAPL) represents one of the largest physical collections of NPs derived from African medicinal plants that is available for screening purposes [5]. This collection was previously screened to identify new inhibitors of HIV and influenza A virus replication as well as inhibitors of sirtuin activity [6–10]. As these studies all demonstrate the ability to obtain novel screening hits from p-ANAPL, we hypothesized that p-ANAPL also likely harbors NPs that may act on SARS-CoV-2 replication.

SARS-CoV-2 entry depends primarily on the binding of the receptor-binding domain (RBD) of the viral Spike protein to the host ACE2 receptor. This interaction facilitates proteolytic activation of Spike by host proteases, which, in turn, initiates membrane fusion and viral entry [11]. Our group and others have identified numerous NPs that selectively interfere with RBD/ACE2 interactions and/or inhibit SARS-CoV-2 replication in live virus or pseudovirus assays [11–16]. We therefore anticipated that NPs from p-ANAPL with activity against SARS-CoV-2 replication would be identified by initial screening for their ability to disrupt RBD/ACE2 protein-protein interactions. Here, we describe the results of this screen, which identified the previously reported oleanolic acid, as well as new leads poinsettifolin B and rhuschalcone III, as inhibitors of RBD/ACE2 binding and/or SARS-CoV-2 replication.

## Materials and methods

### Cells, viruses, and reagents

Vero-E6 cells were obtained from the American Tissue Culture Collection. Cells were cultured in D10 + medium (Dulbecco’s modified Eagle medium with 4.5 g/liter glucose and L-glutamine [Gibco, Gaithersburg, MD, USA], 10% fetal bovine serum [Gemini Bio Products, West Sacramento, CA, USA], 100 U/mL penicillin, and 100 µg/mL streptomycin [Sigma-Aldrich, St. Louis, MO, USA]) in a humidified incubator at 37 °C and 5% CO_2_. The following reagent was deposited by the Centers for Disease Control and Prevention and obtained through BEI Resources, NIAID, NIH: SARS-related coronavirus 2, isolate USA-WA1/2020, NR-52,281. Oleanolic acid, poinsettifolin B, and rhuschalcone III were obtained from p-ANAPL chemical stocks and as described previously [17–19]. (-)-Hopeaphenol was obtained as described previously [12].

### Alphascreen assays

Protein-protein interaction assays were described previously [12]. Briefly, protein–protein interactions were assessed using AlphaScreen technology, where binding of SARS-CoV-2 spike RBD to human ACE2, and of PD-1 to PD-L1, were measured in 384-well white opaque plates under optimized buffer conditions. Recombinant F_c_- and His-tagged proteins (Sino Biological, Chesterbrook, PA, USA) were incubated with donor and acceptor beads in the presence of test compounds dispensed in DMSO. Following 2 h of incubation at room temperature, AlphaScreen signals were recorded on a ClarioStar plate reader (BMG Labtech, Cary, NC, USA). Data were normalized to percentage inhibition, with values expressed relative to positive (absence of His-tagged partner protein) and negative (both proteins plus DMSO vehicle) controls. All conditions were performed in duplicate.

### Viral cytopathic effect quantitative assay

SARS-CoV-2 virus (USA-WA1/2020) was generated, and viral assays were performed in a biosafety level 3 facility as described previously [12]. Briefly, Vero-E6 cells were plated in D10 + to 20,000 cells per well in a 96-well format. Compounds were added to final concentrations and incubated for 2 h before the addition of 150X tissue culture infectious dose 50% (TCID_50_) of virus. Cells were incubated for an additional 4 days, treated with 20 µg/mL resazurin for 4 h (Sigma-Aldrich) to assess cell viability, fixed with paraformaldehyde to a final concentration of 4%, and incubated at room temperature for 30 min. Fluorescence intensity was then measured using a ClarioStar plate reader. Background fluorescence was subtracted from all wells based on the signal obtained from wells containing D10 + and resazurin and no cells. Data were then normalized to the signal obtained from wells containing uninfected cells.

### Cell viability assays in uninfected cells

Assays were performed as described above in the absence of virus. Background fluorescence was subtracted from all wells based on the signal obtained from wells containing D10 + and resazurin and no cells. Data were then normalized to the signal obtained from wells containing cells with no drug treatment.

### Target proteins for docking

All molecular modeling was conducted as previously reported [20–24]. The X-ray crystal structure (PDB ID: 6M0J) describing the RBD of SARS-CoV-2 spike in complex with the ACE2 human receptor protein, corresponding to the parental Wuhan strain, was retrieved from the Protein Data Bank (PDB; [25–27]).

### Protein preparation

Water molecules were first removed using the graphical user interface of the Molecular Operating Environment (MOE) suite [28] (version 2016.08; Chemical Computing Group, Montreal, QC, Canada). Next, the protein preparation for docking was carried out using the Protein Preparation Wizard integrated in the Schrödinger package software [29, 30]. This was done by adding the missing H-bonds, assigning bond orders, and completing the missing side chains. This was followed by energy minimization to remove all atomic clashes and optimize the potential protein-ligand interactions during docking. Epik-tool was then used to predict the protonation states at a pH of 7.0 [31]. Finally, a restrained energy minimization step was conducted using the Optimized Potentials for Liquid Simulations 2005 (OPLS2005) forcefield [32], allowing a root mean square deviation (RMSD) of the displacement of the atoms to end with the minimization at 0.3 Å.

### Ligand preparation

The MOE builder module was used to generate the 3D models of compounds for docking. These were then energy-minimized using the MMFF94 force field [33–37]. Further preparation for docking was done with the LigPrep tool to generate all the plausible tautomers of each ligand, using Schrödinger’s Maestro software package [31] with the incorporated OPLS2005 force field [32] for energy minimization. The ConfGen tool was then used to compute 60 conformers for each ligand, by setting all other options to default except for the minimization of the output [38].

### Docking, scoring, and selection of binding poses

Docking validation and docking of the prepared ligands were carried out using the Glide program incorporated in Schrödinger’s Maestro package [30] and as shown in our recent publications [20–24]. A docking grid box was generated for the RBD/ACE2 complex around the following amino acid residues; Asp597, Thr598, Lys516, Val321, Gln121, Lys578, Ala283, Ser91, Asn746, Gln68, Pro744, Glu518 and Thr610. A total of 10 poses per ligand conformer were taken into consideration, and all other settings were allowed to default. The outputs were scored using the Standard Precision (SP) glide score (GScore) as the scoring function [39]. After the extraction of the docked outputs, the specific area where ligands bound to the RBD/ACE2 complex was observed to identify the residues taking part in the interactions. Docking control of (-)-hopeaphenol was already done in previous studies [23].

### Rescoring by MM-GBSA

The Molecular Mechanics Generalized Born Solvation Area (MM-GBSA) model was employed as a means of re-scoring the selected protein-ligand docked poses by implementing the PRIME tool incorporated in the Maestro package from Schrödinger [29]. The free energy of binding (ΔG_bind_) for each ligand towards the RBD/ACE2 complex was calculated by using the Prime MM-GBSA algorithm with the default settings. Each docked pose was retrieved from the Glide docking output and input into the PRIME program for calculating several thermodynamic properties. The computed properties include the binding free energy (ΔG_bind_) and solvation free energy (ΔG_solv_) values in kcal/mol. The binding pose of the complex structures was visually inspected using the ligand interaction tool in MOE to gain insight into the binding mode of each docked ligand inside the angiotensin II binding site of the ACE2 protein. To investigate the potential of the pANAPL hits acting by alternative mechanisms, including inhibiting other SARS-CoV-2 enzymes, the hits were docked towards the SARS-CoV-2 main protease (M^pro^) with PDB ID: 7L0D and the RNA-dependent RNA polymerase (RdRp, with PDB ID 7BV2) [40, 41].

### Aggregation prediction calculations

Aggregation prediction was conducted for all 3 compounds using the freely available Aggregator prediction webserver available at: https://advisor.bkslab.org [42]. So for each natural product, the SMILES were obtained from PubChem and run at the standard 0.1 to 10 µM concentration.

### Generative modeling

The 3 active compounds were subsequently optimized through de novo design. This generative process began by using their SMILES strings as input to REINVENT4’s mol2mol generator [43–45]. REINVENT4 [46–48] was executed via the command line on an 8-CPU-core Ubuntu 24.04 LTS system. After generation, molecules with invalid SMILES were automatically discarded, and those with valid SMILES were visually assessed; selected molecules proceeded to further evaluation, including docking and MM-GBSA analysis, to ensure proper selection.

## Results

### A subset of p-ANAPL NPs disrupts SARS-CoV-2 RBD / host ACE2 binding

**Fig. 1:**
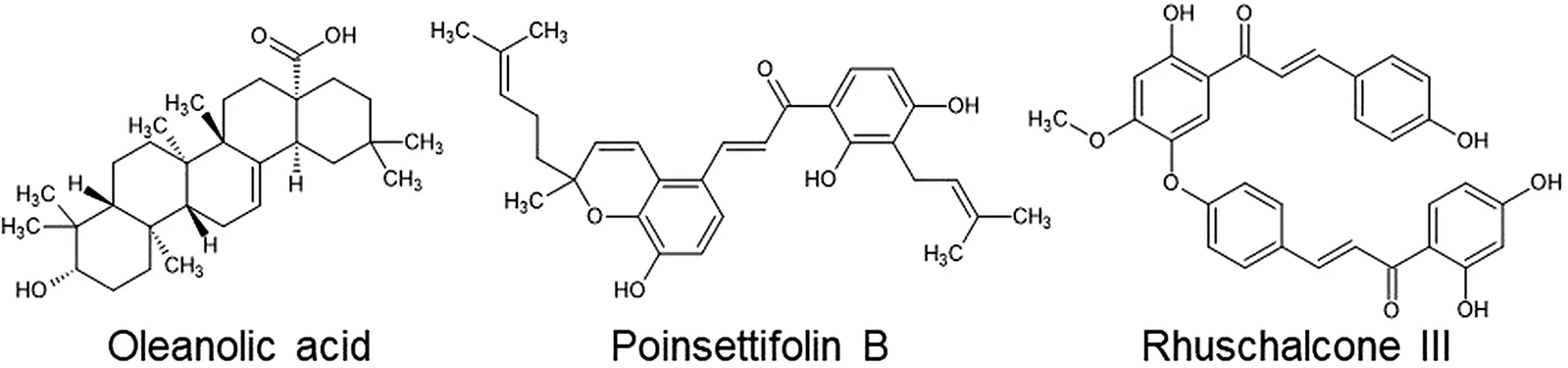
Structures of three NPs isolated from p-ANAPL that disrupt > 80% of RBD/ACE2 binding at 1 µg/mL

To identify NPs with the potential to inhibit viral entry, we used an established binding assay where a SARS-CoV-2 RBD protein (USA-WA1/2020) with a C-terminal His tag, prebound to an acceptor bead, was co-incubated with a full-length ACE2 protein with a C-terminal F_c_ tag, prebound to a donor bead [12, 49]. Following co-incubation, ligand/receptor binding events were measured by excitation at 680 nm, resulting in a single oxygen transfer between donor and receptor beads and luminescence at 615 nm. Using this assay, we then screened 213 readily available physical compounds from p-ANAPL at 1 µg/mL assembled as part of a previous study to identify natural product-based HIV latency reversing agents [9]. Three compounds were identified to inhibit at least 80% of RBD/ACE2 binding-dependent luminescence; these compounds included oleanolic acid, poinsettifolin B, and rhuschalcone III (Fig. 1; Table 1). An additional seven compounds also inhibited binding between 70 and 80% (Table 1). Given the number of NPs with the potential to disrupt RBD/ACE2 binding, we prioritized the three most active compounds for study, including assessment against primary virus replication in biosafety level 3 conditions.

**Table 1:** Compounds from p-ANAPL that inhibit at least 70% of SARS-CoV-2 Spike RBD/ACE2 binding at 1 µg/mL

Compound	Percent inhibition
Oleanolic acid	83.5
Poinsettifolin B	81.7
Rhuschalcone III	80.8
4-Hydroxylonchocarpin	78.0
Agathisflavone	76.9
Pelargonidin chloride	76.3
4’-Demethylknipholone	75.5
Knipholone 6-methylether	72.2
Trans-Hinokiresinol	70.6
6,8-Diprenyleeriodictyol	70.4

**Fig. 2:**
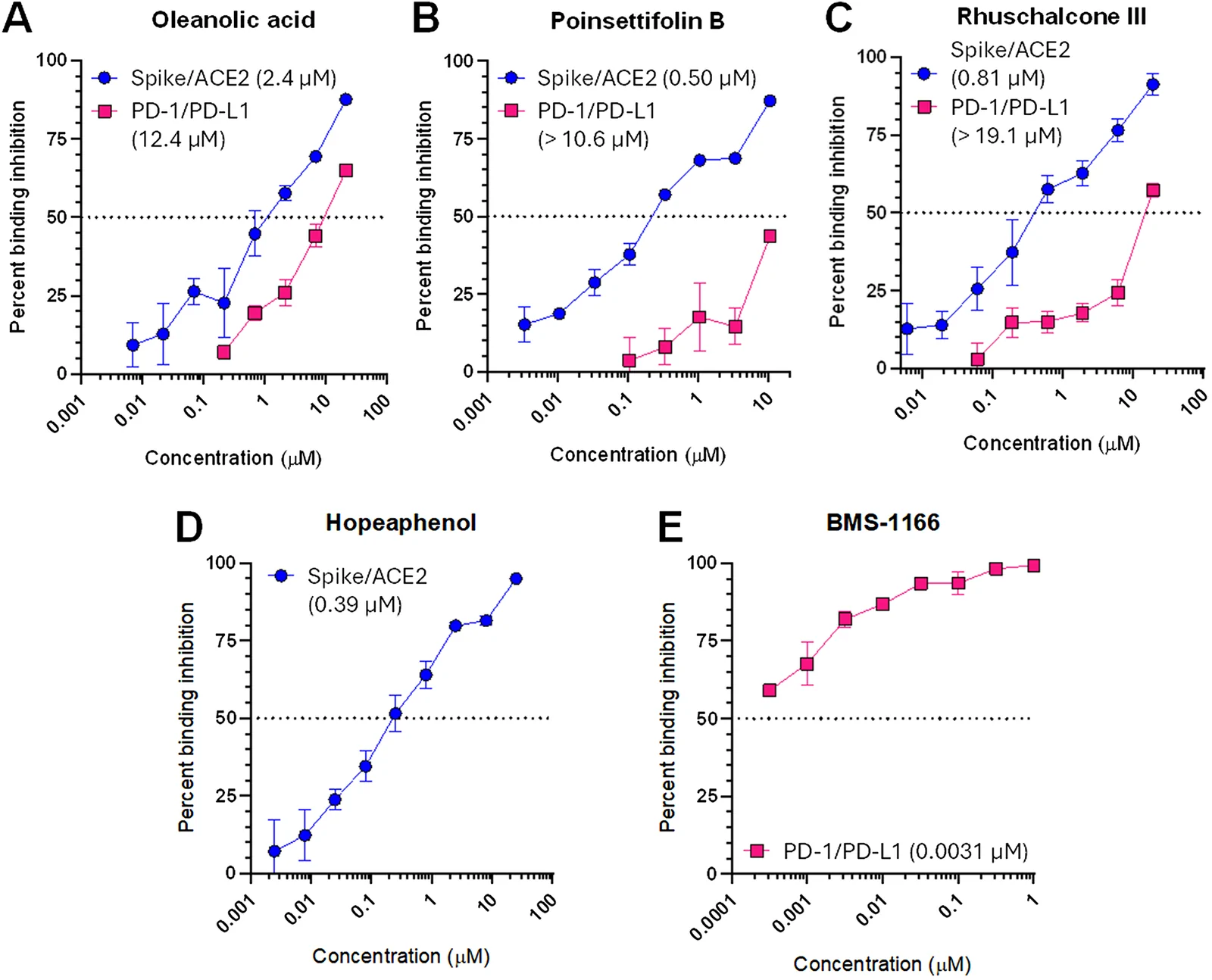
Dose-response curves of oleanolic acid (**A**), poinsettifolin B (**B**), rhuschalcone III (**C**), and control inhibitors hopeaphenol (**D**) and BMS-1166 (**E**), indicating ability to disrupt binding of RBD and ACE2 (blue) and/or PD-1 and PD-L1 proteins (red). For each panel, values in parenthesis denote respective IC_50_s

We next assessed the relative activity of these three compounds to disrupt RBD/ACE2 binding by repeating the binding assay in dose-response studies [12]. Dose-response curves were obtained for all three compounds, allowing us to calculate half-maximal inhibitory concentrations (IC_50_s) of 2.4, 0.50, and 0.81 µM for oleanolic acid, poinsettifolin B, and rhuschalcone III, respectively (Fig. 2A-C). In contrast, the previously described positive control inhibitor (-)-hopeaphenol [12] disrupted binding in this assay with an IC_50_ of 0.39 µM (Fig. 2D). Thus, the three compounds identified here are roughly 1.3 to 6.2-fold less active than this established disruptor of RBD/ACE2 binding.

To determine whether these compounds may selectively disrupt RBD/ACE2 binding over other protein-protein interactions, we repeated the binding assay using the unrelated host PD-1/PD-L1 ligand/receptor pair [12]. While the positive control inhibitor BMS-1166 [50] disrupted PD-1/PD-L1 binding in this assay with an estimated IC_50_ of 0.0031 µM (Fig. 2E), oleanolic acid, poinsettifolin B, and rhuschalcone III had substantially weaker activities, with IC_50_s of 12.4, > 10.6, and > 19.1 µM, respectively (Fig. 2). When comparing the ratios of IC_50_s of PD-1/PD-L1 and RBD/ACE2 binding for the three compounds, we observed selectivity indices of 5.2 for oleanolic acid, > 21.4 for poinsettifolin B, and > 23.5 for rhuschalcone III, indicating that all three NPs selectively disrupt RBD/ACE2 binding.

### p-ANAPL NPs interact with amino acid residues required for SARS-CoV-2 RBD and host ACE2 binding

To identify the mechanisms by which oleanolic acid, poinsettifolin B, and rhuschalcone III might disrupt the RBD/ACE2 complex, molecular docking was performed by targeting the angiotensin II binding site within the complex. This was followed by a rescoring step to estimate the binding free energy ΔG_bind_ of the resulting ligand–receptor complexes. The docking scores obtained for oleanolic acid, poinsettifolin B, and rhuschalcone III were − 4.312 kcal/mol, − 4.703 kcal/mol, and − 6.000 kcal/mol, respectively (Table 2). Accordingly, the predicted binding affinities for these ligands toward the RBD/ACE2 complex followed the order of rhuschalcone III > poinsettifolin B > oleanolic acid. The major components of the Glide docking score, namely the van der Waals (GScore_vdw_) and electrostatic (GScore_ele_) contributions, are presented in Table 2. For rhuschalcone III, poinsettifolin B, and oleanolic acid, GScore_vdw_ and GScore_ele_ were − 38.585 and − 16.285 kcal/mol, -38.583 and − 5.516 kcal/mol, and − 31.576 and − 7.815 kcal/mol, respectively. In all cases, van der Waals interactions contribute more substantially to the total docking score than electrostatic interactions, suggesting that hydrophobic and steric complementarity may play a predominant role in the binding of these compounds to the RBD/ACE2 interface. Interestingly, rhuschalcone III exhibited a significantly higher electrostatic contribution compared to the other two compounds, which may indicate a greater involvement of polar or charged interactions in its binding mode. Rescoring of the docked complexes further supported the docking score trend, with calculated ΔG_bind_ values of − 29.29, − 34.37, and − 42.29 kcal/mol for oleanolic acid, poinsettifolin B, and rhuschalcone III, respectively (Table 2). The solvation energy contributions (ΔG_solv_) were calculated to be + 33.911, + 37.503, and + 32.360 kcal/mol for rhuschalcone III, poinsettifolin B, and oleanolic acid, respectively (Table 2). These positive values reflect the fact that, in the unbound state, solvation significantly stabilizes the charged or polar groups of both the ligand and the receptor through interactions with water molecules. Upon complex formation, this stabilization is reduced, resulting in a positive ΔG_solv_. Notably, the solvation energies of rhuschalcone III and oleanolic acid are nearly identical and more favorable than that of poinsettifolin B, suggesting a lower desolvation penalty for these two compounds during binding.

**Table 2:** Docking (GScore) score and ΔG_bind_ (Prime MM-GBSA) values of NPs with the SARS-CoV-2 RBD/ACE2 complex

Complexes	Docking score (kcal/mol)	GScore_ele_	GScore_vdW_	ΔG_bind_ (kcal/mol)
6M0J/oleanolic acid	-4.312	-31.576	-7.815	-29.29
6M0J/poinsettifolin B	-5.421	-38.583	-5.516	-30.48
6M0J/rhuschalcone III	-6.00	-39.585	-16.285	-42.29

**Fig. 3:**
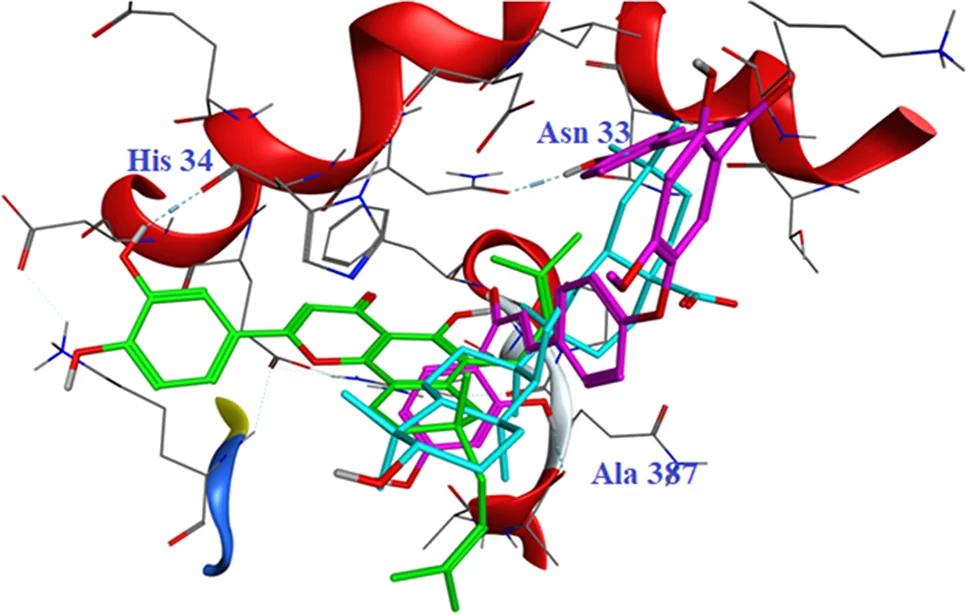
Superposition of three NPs within the binding site of the RBD/ACE2 complex following molecular docking. Oleanolic acid is shown in cyan, poinsettifolin B is in green, and rhuschalcone III is in violet

We next analyzed the binding conformations of the three ligands to the RBD/ACE2 complex directly within the active site. Figure 3 illustrates the superimposed structures and spatial orientation of oleanolic acid, poinsettifolin B, and rhuschalcone III within the binding site of the complex using MOE software. While oleanolic acid and rhuschalcone III occupy a similar region of the binding pocket, rhuschalcone III forms two key hydrogen bonds with residues Ala387 and Asn33 of the 6M0J protein complex, while no hydrogen bonds are observed with oleanolic acid. In contrast, poinsettifolin B, perhaps due to greater conformational flexibility, partially occupies the same region as the other two ligands but also extends into an adjacent sub-pocket where it forms a key hydrogen bond with residue Gln37 of the ACE2 site of the 6M0J complex. Interestingly, the number of hydrogen bonds by each ligand also correlates with their docking scores and ΔG_bind_ values (Table 2).

**Fig. 4:**
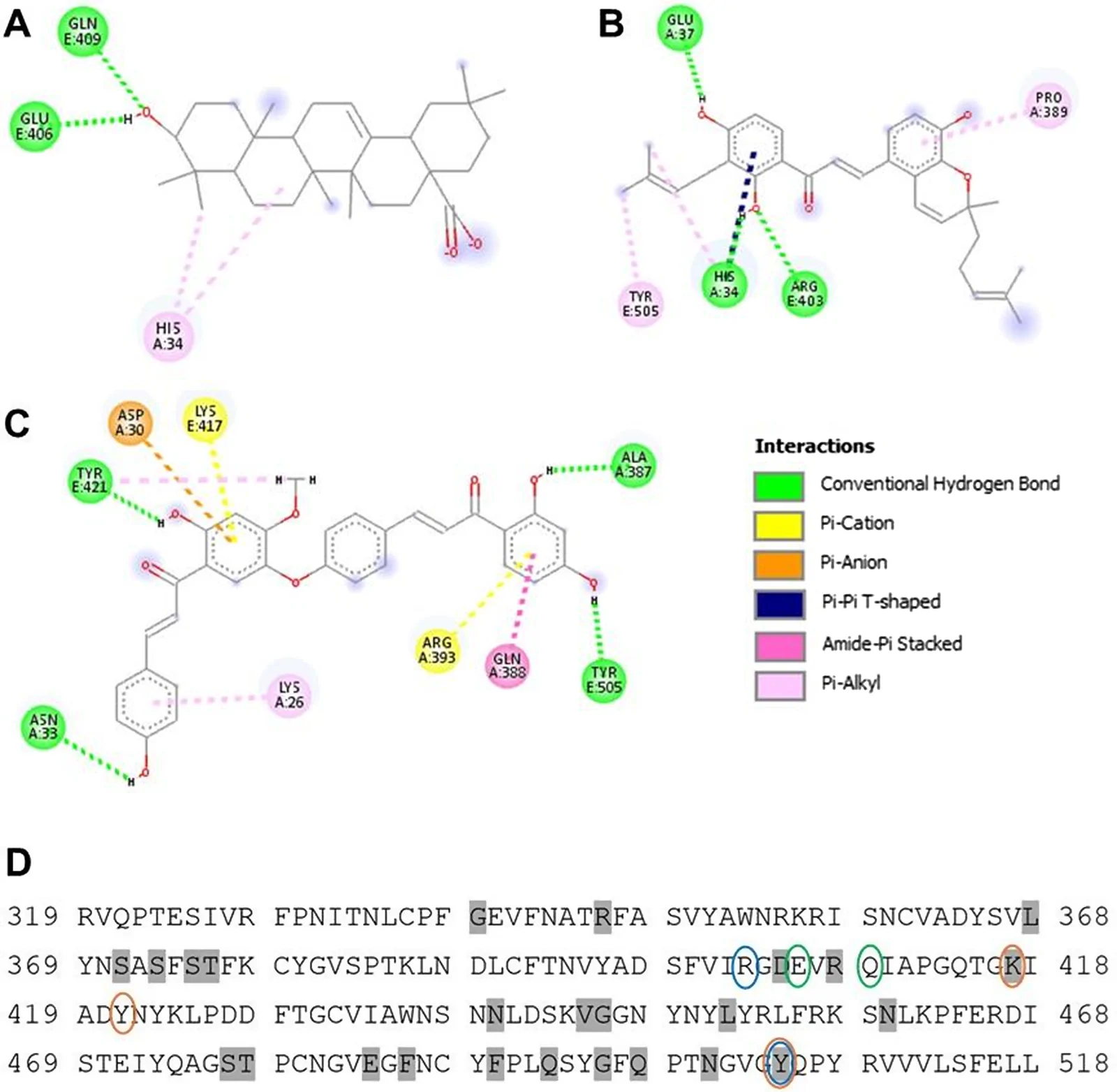
2D interaction diagrams of oleanolic acid (**A**), poinsettifolin B (**B**), and rhuschalcone III (**C**) within the binding site of the spike RBD/ACE2 complex. **D**, Summary of binding contacts of three NPs within the canonical sequence of the parental SARS-CoV-2 Spike RBD (USA-WA1/2020). Interactions by oleanolic acid, poinsettifolin B, and rhuschalcone III with the RBD are highlighted in cyan, green, and orange, respectively. Sequence changes in variants of concern are highlighted in gray

To investigate further the specific interactions between each ligand and the SARS-CoV-2 RBD/host ACE2 complex, we next generated 2D interaction diagrams using Discovery Studio. As shown in Fig. 4A, oleanolic acid forms two conventional hydrogen bonds with Gln409 and Gln406 in addition to a single π–alkyl interaction with His34 of the ACE2 site of the 6M0J complex. The 2D interaction diagram of poinsettifolin B also reveals stabilization through three conventional hydrogen bonds involving residues His34, Arg403, and Gly37, along with a π–π T-shaped interaction with His34, and π–alkyl interactions with Tyr505, Pro389, and His34 (Fig. 4B). In contrast, rhuschalcone III forms four conventional hydrogen bonds with residues Asn33, Ala387, Tyr421, and Tyr505. In addition, two π–cation interactions are observed with Arg393 and Lys417. Other notable interactions include a π–anion interaction with Asp30, an amide–π stacking interaction with Gln388, and a π–alkyl interaction with Lys26 (Fig. 4C). These results are also consistent with rhuschalcone III having the strongest binding affinity with the RBD/ACE2 complex through the highest number and diversity of specific interactions with key residues in the binding site.

When the binding sites of each compound were overlayed with the canonical RBD protein sequence, we found that oleanolic acid interactions with RBD did not overlap with mutations arising in multiple SARS-CoV-2 variants including Alpha (B.1.1.7), Beta (B.1.351), Delta (B.1.617.2) and Omicron (B.1.1.529.1, BA.1, BA.2, and XBB.1.5). In contrast, poinsettifolin B interacted with Tyr505, which is mutated to His in Omicron variants, while rhuschalcone III interacted with both Tyr505 and Lys417, which is additionally mutated to Asn in both Beta and Omicron variants (Fig. 4D).

The observations of selective RBD/ACE2 disruption and detectable antiviral activity by rhuschalcone III led us to ask whether additional bichalcones of this large chemical family may also target the SARS-CoV-2 RBD/ACE2 interaction. To investigate this, we searched the PubChem repository for analogues exhibiting ≥ 95% structural similarity to the parent scaffold rhuschalcone III. This search yielded 132 candidate molecules, which were subjected to molecular docking under identical experimental conditions as the reference rhuschalcone III. Table 3 reports the docking outcomes for the top 12 scoring molecules from this collection. From these results, one observes that the docking scores of the selected twelve analogues span from − 6.024 to − 7.495 kcal/mol, compared to the docking score of the parent rhuschalcone III of − 6.00 kcal/mol (Table 2). Thus, all twelve analogues exhibit more favorable predicted binding affinities relative to rhuschalcone III. Further dissection of the major energy-terms reveals that the van der Waals component (GScore_vdw_) ranges from − 34.448 to − 45.336 kcal/mol, whereas the coulombic component (GScore_ele_) spans − 4.110 to − 18.201 kcal/mol.

These data indicate that van der Waals (i.e., hydrophobic/steric) interactions markedly dominate over electrostatic (Coulomb) contributions for all compounds. Notably, this trend was also observed with the reference rhuschalcone III, suggesting that hydrophobic and steric complementarity may play a predominant role in the binding of these compounds to the RBD/ACE2 interface. The resulting complexes were further re-scored using the MM-GBSA approach. The calculated binding free energies (ΔG_bind_) varied from − 36.703 to − 52.311 kcal/mol across the different analogues. Except for the two compounds registered under PubChem IDs 170221756 and 146036329, all molecules exhibited more favorable ΔG_bind_ values than that of rhuschalcone III (− 42.29 kcal/mol). The solvation energy contribution ranged from + 21.895 to + 43.037 kcal/mol for the various complexes; these positive values, as seen also for rhuschalcone III, are consistent with a binding mode dominated by hydrophobic (i.e., desolvation/van der Waals) rather than polar interactions. Taken together, these findings strongly support the hypothesis that analogues within the rhuschalcone III family may constitute promising inhibitors of the SARS-CoV-2 spike RBD/ACE2 complex. Table 4 summarizes the docking scores and ΔG_bind_ (Prime MM-GBSA) values of the pANAPL hits towards the binding sites of two other SARS-CoV-2 targets: M^pro^ (PDB ID: 7L0D) and RdRp (PDB ID:7BV2), with a view of investigating whether they are likely to bind as well to other SARS-CoV-2 targets and act via different mechanisms.

**Table 3:** Docking (GScore) score and ΔG_bind_ (Prime MM-GBSA) values for analogues exhibiting ≥ 95% structural similarity to the parent scaffold rhuschalcone III toward the SARS-CoV-2 spike RBD/ACE2 complex

Compounds (Pubchem ID)	Docking score (kcal/mol)	GScore_ele_ (kcal/mol)	GScore_vdw_ (kcal/mol)	ΔG_bind_ (kcal/mol)	ΔG_solv_ (kcal/mol)
170221756	-7.495	-16.388	-41.448	-36.836	39.572
13873817	-6.642	-11.028	-40.496	-52.311	32.504
13963933	-6.637	-8.429	-40.146	-48.614	30.961
118898656	-6.516	-10.908	-39.159	-49.981	32.289
173625139	-6.516	-10.908	-39.159	-49.981	32.289
163103777	-6.365	-18.201	-36.598	-44.766	40.004
162966847	-6.217	-10.216	-39.451	-46.213	43.037
10816879	-6.210	-5.267	-45.338	-47.241	38.689
142830944	-6.175	-4.796	-41.924	-48.102	29.427
404554	-6.168	-16.619	-36.871	-46.404	40.818
5472700	-6.168	-16.619	-36.871	-46.404	40.818
146036329	-6.024	-4.110	-34.448	-36.703	21.895

**Table 4:** Docking scores and ΔG_bind_ (Prime MM-GBSA) values of panel compounds towards the binding sites of two other SARS-CoV-2 targets: M^pro^ (PDB ID: 7L0D) and RdRp (PDB ID:7BV2)

Compound	SARS-CoV-2 Spike/ACE2 (PDB ID: 6M0J)		SARS-CoV-2 M^pro^ (PDB ID: 7L0D)		SARS-CoV-2 RdRp (PDB ID: 7BV2)	
	Docking score (kcal/mol)	ΔG_bind_ (kcal/mol)	Docking score (kcal/mol)	ΔG_bind_ (kcal/mol)	Docking score (kcal/mol)	ΔG_bind_ (kcal/mol)
oleanolic acid	-4.312	-29.29	-4.764	-27.59	-2.716	-17.00
poinsettifolin B	-5.421	-30.48	-6.527	-53.47	-4.062	-11.67
rhuschalcone III	-6.000	-42.29	-8.015	-51.14	-4.990	-29.25
7L0D/ML188	-	-	-6.272	-74.81	-	-
7BV2/Remdesivir triphosphate	-	-	-	-	-6.481	-11.23

### Aggregation prediction for selected p-ANAPL hits

The results for the prediction have been summarized in Table 5, showing that none of the NPs are predicted to provoke aggregation of solutions in assays. Besides, the closest (most similar) known aggregators to these NPs show Tanimoto similarity coefficients (*T*_*c*_) of less than 0.5, except for the similar compound to poinsettifolin B (i.e., **MLS000574837-01**), with a T_*c*_ of 0.54.

**Table 5:** Aggregation prediction for selected p-ANAPL hit compounds using

Compound	Predicted aggregator	Similar aggregating agent(s)	*T*_*c*_	Reference
Oleanolic acid	No	**NCGC00095094-01**	0.48	[42]
rhuschalcone III	No	**NCGC00142412-01**	0.47	
		**MLS000575680-01**	0.47	
		**NCGC00095321-01**	0.46	
		**MLS000574837-01**	0.46	
poinsettifolin B	No	**MLS000574837-01**	0.54	

### p-ANAPL NPs inhibit in vitro SARS-CoV-2 replication without cellular toxicity

To confirm whether the disruption of RBD/ACE2 binding by these NPs corresponded to the ability to inhibit virus replication, we infected Vero-E6 cells with SARS-CoV-2 virus (USA-WA1/2020) in the presence or absence of compounds and monitored cellular viability after 96 h using a previously established multicycle virus replication assay [12].

**Fig. 5:**
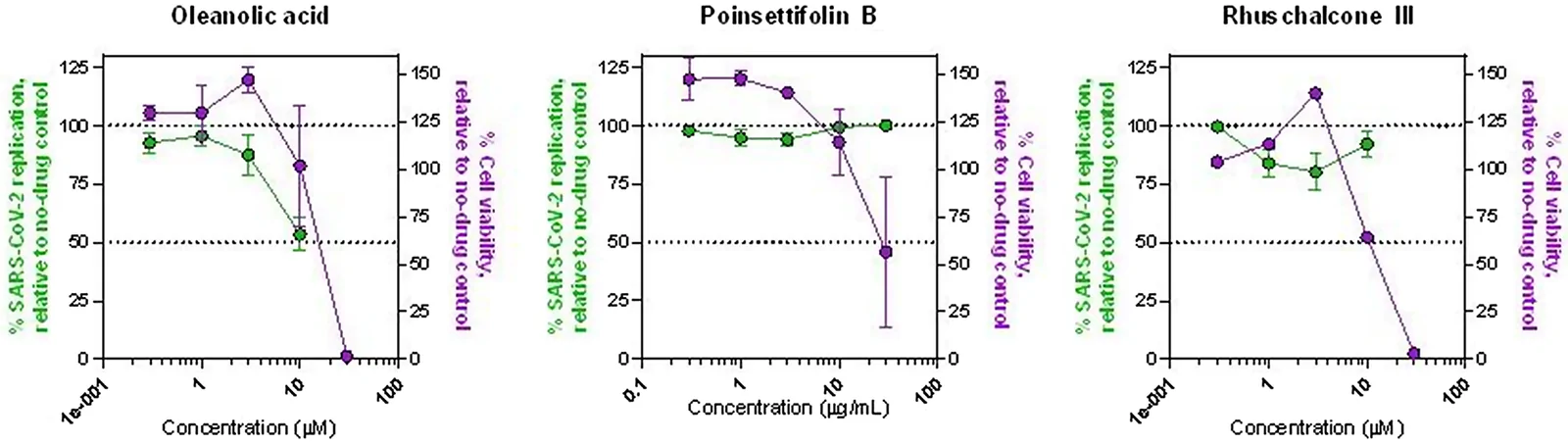
Dose-response curves of oleanolic acid (**A**), poinsettifolin B (**B**), and rhuschalcone III (**C**) indicating ability to inhibit SARS-CoV-2 replication in infected Vero cells (green) and cell viability in uninfected Vero cells (violet). In these experiments, control inhibitor remdesivir inhibited 85.8 ± 11.4% replication at 10 µM, consistent with its previously reported EC_50_ of 7.3 µM in this assay [12]

In parallel, we also treated uninfected Vero cells with compounds and measured viability after 96 h to assess the intrinsic cytotoxicity of each NP. Of the three compounds, we observed that oleanolic acid was the most active, with 46.6 ± 7.1% (mean ± s.e.m.) block of virus replication at 10 µM, relative to infected cells without drug treatment (Fig. 5A), and indicating as a result an estimated half-maximal effect concentration (EC_50_) of 10.8 µM. This activity approached that of the positive control inhibitor remdesivir, which inhibited 85.8 ± 11.4% of virus replication at 10 µM, which in turn is consistent with its previously reported EC_50_ of 7.3 µM by us in this assay [12]. However, the antiviral activity of oleanolic acid at higher concentrations could not be determined due to off-target cytotoxicity caused by the compound, where we calculated a half-maximal cytotoxic concentration (CC_50_) of 19.9 µM (Fig. 5A), indicating 1.8-fold selectivity for antiviral activity over toxicity. In contrast, while poinsettifolin B did not inhibit SARS-CoV-2 replication at any concentration but did display cytotoxicity at concentrations that might mask its antiviral activity (CC_50_ = 10.8 µM; Fig. 5B), rhuschalcone III exhibited weak but consistent activity where, for example, it inhibited up to 19.9 ± 7.7% of SARS-CoV-2 replication at 3 µM, although less activity was seen at higher concentrations, possibility due to increased intrinsic cytotoxicity of this NP (CC_50_ = 9.3 µM; Fig. 5C). Taken together, these results indicate that oleanolic acid, and to a lesser extent, rhuschalcone III, have antiviral activity against SARS-CoV-2 in addition to their ability to disrupt RBD/ACE2 binding.

### Generative modeling of p-ANAPL hits

**Fig. 6:**
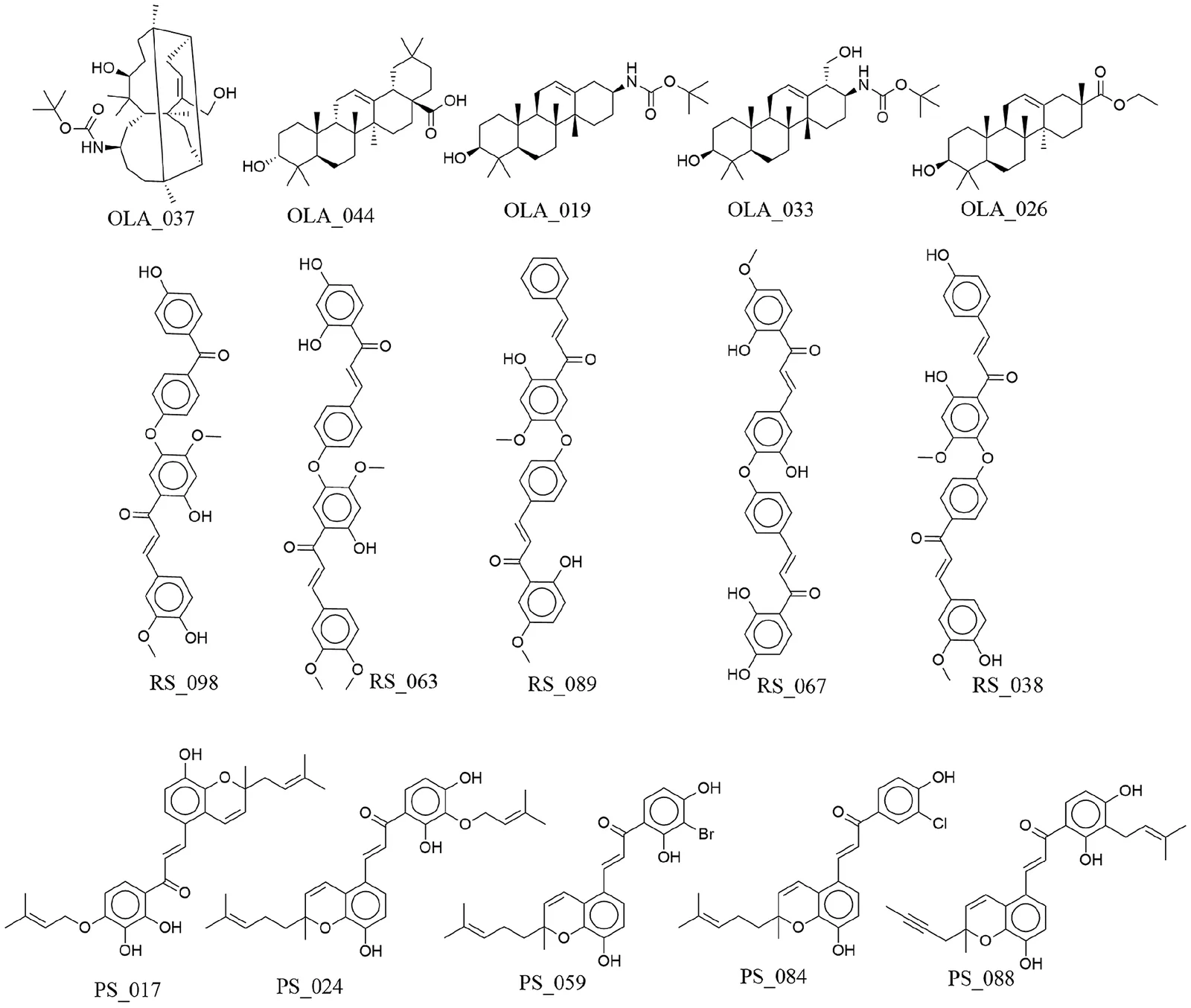
Structures of the top de novo-generated hits based on MM-GBSA re-scoring

While rhuschalcone III and poinsettifolin B had limited or no ability, respectively, to inhibit in vitro SARS-CoV-2 replication, their ability to selectively inhibit RBD/ACE2 interactions at sub-micromolar concentrations warrant consideration of designing and testing analogues with, for example, potentially improved stability in solution, target engagement in a cellular context, improved cell permeability, and/or reduced cytotoxicity or other off-target effects. Figure 6 shows selected virtual (de novo)-designed hits, derived from generative modeling, having lower binding free energy values than the 3 active compounds from the p-ANAPL library. Five analogs have been selected per compound and the docking scores and ΔG_bind_ values have been summarized in Table 6.

**Table 6:** Docking scores and ΔG_bind_ values of the top de novo-generated hits compared to the NP hits

Compound code	docking score (GScore)	ΔG_bind_ (kcal/mol)
OLA_037	-4.012	-36.47
OLA_044	-3.809	-36.18
OLA_019	-4.974	-36.16
OLA_033	-4.254	-33.44
OLA_026	-4.812	-31.48
Oleanolic acid	**-4.312**	**-29.29**
RS_098	-7.04	-64.18
RS_063	-7.382	-55.8
RS_089	-6.226	-55.41
RS_067	-6.27	-54.7
RS_038	-6.425	-53.09
Rhuschalcone III	**-6.00**	**-42.29**
PS_017	-5.74	-52.16
PS_024	-3.959	-45.97
PS_059	-5.242	-45.28
PS_084	-5.089	-45.27
PS_088	-4.496	-44.79
Poinsettifolin B	**-5.421**	**-30.48**

## Discussion

Here we probed 213 compounds from the pan-African Natural Products Library, a collection obtained from African medicinal plants for screening purposes, to identify NPs that disrupt SARS-CoV-2 spike RBD / host ACE2 protein interactions and/or SARS-CoV-2 replication in vitro. The three most active NPs – oleanolic acid, poinsettifolin B, and rhuschalcone III – all disrupted RBD/ACE2 interactions at low or sub-micromolar activities with selectivity over the unrelated PD-1/PD-L1 protein-protein interaction. Oleanolic acid and rhuschalcone III, but not poinsettifolin B, also inhibited SARS-CoV-2 replication in infected Vero cells at concentrations that did not induce substantial cytotoxicity. These compounds represent useful biological probes for elucidating detailed mechanisms of disrupting SARS-CoV-2 entry and replication, for example through use of recombinant pseudoviruses, time-of-addition viral studies, and/or direct binding validation using surface plasmon resonance or related technologies, as well as structural leads that can serve as the basis for chemical derivatives with improved preclinical properties against SARS-CoV-2 and potentially other coronaviruses when compared to existing therapies like remdesivir.

Oleanolic acid is a triterpene compound found in many fruits and medicinal plants and is best characterized by its antioxidant and anti-inflammatory properties [51, 52]. It has multiple total synthesis methods and natural and synthetic derivatives [51], making it a promising starting scaffold for future drug development. It is also previously reported to interact with both RBD and ACE2 [53, 54], although the RBD and ACE2 residues bound by oleanolic acid in these studies differ from ours and from each other, suggesting multiple binding poses that can confer its mechanism of action. It was also found to disrupt entry of SARS-CoV-2 spike-expressing pseudoviruses as well as in vitro SARS-CoV-2 replication at low micromolar concentrations [55]. Our study supports these previous observations and further shows that oleanolic acid has selectivity for RBD/ACE2 disruption over unrelated ligand-receptor interactions. It also validates our strategy to identify new anti-SARS-CoV-2 agents from NP libraries.

On the other hand, this is the first report describing poinsettifolin B and rhuschalcone III as SARS-CoV-2 inhibitors. Poinsettifolin B is a chalcone-type flavonoid originally isolated from *Dorstenia poinsettifolia*, an herb found in forests of Cameroon and Gabon and reported to be traditionally used to treat yaws and infected wounds [18, 56, 57]. While it has reported cytotoxicity in some cell lines [58], its bioactivities, as well as those of its few known analogs, are largely unknown. In contrast, rhuschalcone III is a bichalcone originally isolated from the root bark of *Searsia pyroides* (previously named *Rhus pyroides*), which is traditionally used in South Africa to manage epilepsy [19, 59]. While extracts of *S. pyroides* have in vitro activities consistent with gamma-aminobutyric acid agonists and/or NMDA antagonists [59, 60], these activities have not been established for rhuschalcone III. However, we previously reported that rhuschalcone IV and other bichalcone analogues can inhibit sirtuins [7], indicating that this family of compounds may have important therapeutic potential for multiple diseases and human disorders. Taken together, our observation that both poinsettifolin B and rhuschalcone III are selective inhibitors of RBD/ACE2 binding and/or SARS-CoV-2 replication assigns new bioactive properties to these compounds. In addition, the identification of multiple rhuschalcone III analogs with predicted favorable binding affinities against the RBD/ACE2 complex suggests that rhuschalcone III itself could serve not only as a direct inhibitor of RBD/ACE2 binding but also as a scaffold for the rational design of novel anti-COVID-19 agents.

Binding confirmations and 2D interaction diagrams indicate that all three compounds interact at least in part with residues in the RBD that have not undergone substantial mutation from the parental Wuhan sequence to subsequent variants of concern. In particular, oleanolic acid does not interact with any residue with mutations in variants, indicating that it is likely to continue to maintain antiviral activity during RBD sequence evolution over time. This is supported by a study indicating that oleanolic acid continues to inhibit cellular entry of pseudoviruses bearing Beta mutations in the RBD, as well as pseudoviruses with spike sequences from SARS-CoV-1 and MERS-CoV [55]. In contrast, the predicted interactions of poinsettifolin B and rhuschalcone III with Tyr505 and/or Lys417 suggest that these compounds may have reduced ability to disrupt RBD/ACE2 interactions from subsequent SARS-CoV-2 variants of concern, although this hypothesis remains to be tested.

The docking and MM-GBSA analyses of compounds exhibiting 95% structural similarity to rhuschalcone III revealed that many members of this chemical family might effectively disrupt the SARS-CoV-2 RBD/ACE2 complex. Almost all selected analogues showed more favorable docking scores and binding free energies than the parent rhuschalcone III, suggesting enhanced affinity and thermodynamic stability. Energy decomposition indicated that van der Waals interactions predominated over electrostatic contributions, implying that hydrophobic and steric complementarity are key determinants of binding at the RBD/ACE2 interface. These findings are consistent with previous studies reporting flavonoid derivatives, such as quercetin and curcumin analogues, as effective RBD/ACE2 interaction inhibitors [61, 62]. Overall, rhuschalcone III and its close analogues represent promising scaffolds for the rational design of small-molecule inhibitors targeting viral entry.

Compared with the other SARS-CoV-2 enzymes, the docking scores and binding affinity values towards M^pro^ and RdRp in Table 4 showed that these hits may interact strongly with M^pro^ compared to RdRp driven mostly by van der Waals interactions. The analysis was done compared to the respective co-crystallised ligand in each protein target (ML188 for M^pro^ and Remdesivir triphosphate for RdRp). The ΔG_bind_ values suggest that the NP hits have higher affinity to M^pro^ (-51.14 to -27.59 kcal/mol) than RdRp (-29.25 to -17.00 kcal/mol). This suggests that the NP hits inhibit viral replication through another mechanism.

Natural products can sometimes show apparent biological activity due to colloidal aggregation and related nonspecific effects e.g. Curcumin. Therefore, an assessment of the aggregation propensity of the three selected compounds was performed computationally and the analysis demonstrated that the observed activity is less likely to arise from nonspecific aggregation.

The REINVENT4 mol2mol generator employs molecules similar to the input molecules based on medium or high Tanimoto similarity, and then automatically removes invalid structures, i.e., chemically incorrect compounds, synthetically non-tractable, and non-druglike. After filtering these out, the proposed analogs from REINVENT were then ranked based on the computed ΔG_bind_ values derived from PRIME MM-GBSA. For the oleanolic acid, we can observe that the major suggested changes for the analog with the lowest ΔG_bind_ value (**OLA_037**) result from the reduction of the number of rings to only 3 rings and the introduction of a *tert-*butylated amide functionality to increase the number of H-bonds possible at the docking site. **OLA_019** and **OLA_033** both show the same functionality at different positions, while **OLA_026** shows an ester substituent at the same position, with a reduction of the number of rings to 4, as in its predecessors. This implies one way of reducing oleanolic acid to become medicinal chemistry amenable is by the reduction of the number of rings and the introduction of polar moieties.

With respect to Rhuschalcone III, the proposed analogs show that the biphenyl ether moiety is kept intact throughout for the analogs with the lowest predicted binding affinities. The substitutions are proposed on the terminal phenol groups that make the parent molecule toxic. These have been proposed to be methylated to give the equivalent methoxy substitutions, while the caffeol moiety remains unchanged in most cases, except for **RS_098**, where it has been proposed that this be replaced by a keto group.

For the Poinsettifolin B analogs, suggestions include the introduction of an O-atom before the prenyl, thereby increasing the chances of H-bond formation. This is the case with **PS-017** and **PS-024**. Other suggestions include the removal of the bulkier and more hydrophobic prenyl group, while smaller hydrophobic halogen atoms like Cl and Br. In the case of **PS_017**, position isomers of the dihydroxy-substituted phenone moiety from *ortho*-*para* to *ortho*-*meta*, thereby placing the bulkier prenyl substituent at the *para* position, where it could address hydrophobic amino acid residues like Phe486 from the RBD of SARS-CoV-2 spike.

Limitations of the current study include the fact that the X-ray structure used does not include a co-crystallized or reference ligand for the Spike-ACE2 target (6M0J), which is essential for re-docking or docking validation. However, recently published studies show that the key interacting amino acid residues that explained the structure-activity relationships in our recent studies [20–23] are also clearly present in this study. The docking studies could carefully explain the in vitro data, with no further need to carry out molecular dynamics studies to test the stability of the docked protein-ligand complexes. Other limitations include the fact that the in vitro antiviral assays were conducted in Vero cells and, therefore, may not fully reflect human airway infection. Computational docking and analogue generation remain predictive and require experimental validation. 

## Conclusions

In summary, we demonstrate that NPs obtained from African medicinal plants continue to be a rich source of novel antivirals, including against disruption of factors involved in SARS-CoV-2 cellular entry and/or viral replication. Three compounds identified from screening the p-ANAPL here (oleanolic acid, poinsettifolin B, and rhuschalcone III) represent new chemical leads to develop future therapies as well as to further characterize medicinal plants, which may represent locally available options to manage SARS-CoV-2 and potentially future CoV infections in the absence of accessible Western vaccines and therapies. Further biochemical, virological, and structural studies are warranted to validate their mechanisms of action and confirm their activity against contemporary and emerging SARS-CoV-2 variants. The identified scaffolds, together with the computationally designed analogues, provide starting points for medicinal chemistry optimization aimed at improving potency, selectivity, pharmacokinetic properties, and safety. More broadly, these findings highlight the value of systematically exploring African biodiversity and natural-product chemical space for antiviral discovery and pandemic preparedness. Future studies integrating experimental validation with computational approaches may facilitate the development of accessible antiviral candidates with activity against SARS-CoV-2 and potentially other coronaviruses.

## Data Availability

All data generated or analysed during this study are included in this published article.

## Abbreviations

ACE2: Angiotensin-converting enzyme 2 receptor
MM-GBSA: Molecular mechanics generalized born surface area
p-ANAPL: pan-African natural products library
RBD: Receptor binding domain
SARS-CoV-2: Severe acute respiratory syndrome coronavirus 2
